# Scanpath EEG dynamic, a new perspective for neuroaesthetic connoisseurship in paintings

**DOI:** 10.3389/fpsyg.2025.1442623

**Published:** 2025-08-26

**Authors:** Guy Cheron, Jan De Maere

**Affiliations:** ^1^Laboratory of Neurophysiology & Movement Biomechanics (LNMB), Université Libre de Bruxelles (ULB), Brussels, Belgium; ^2^University of Art & Design, Cluj-Napoca, Romania

**Keywords:** scanpaths, neuroaesthetics, art, oculomotricity, visual search, EEG, oscillation, connoisseurship

## Abstract

The analysis of ocular scanpaths during the observation of artistic pictures has paved the way for neuroaesthetics to question the involvement of brain mechanisms during artistic experiences. In this review, we revisit the main aspects of three fundamental domains of investigation implicated in the perception of art and beauty: (1) oculomotor science, (2) vision, and (3) the dynamics of brain oscillations. For each of these fields, central elements are highlighted to demonstrate their functional inter-dependency for the future development of neuroaesthetics, upon which connoisseurship expertise depends. Namely, the scanpath theory, linked to basic neurophysiological concepts such as saccadic and blink suppression, fixational eye movements, and sensorimotor mnemonic, were described and integrated with other important elements of visual search. The meaning, saliency, and integrated priority maps were discussed in relation to working memory and consciousness. Then, the basic and specialized networks of the visual framework were reviewed in relation to bottom-up, top-down, and corollary discharge mechanisms. Finally, the EEG dynamics of alpha and gamma oscillations were proposed to decipher the involvement of brain wave generators during scanpath artistic exploration.

## 1 Introduction

Since the remarkable monograph published by Zangemeister and Stark (2007) devoted to the importance of the theory of ocular scan paths in the dynamics of the artistic brain, the present perspective proposes to trace the new major discoveries in this field to develop new tools for analyzing cerebral activity associated with eye movements during the exploration of art and masterpieces.

In this context, the connoisseurship is an ability of critical quality assessment by the most talented experts with sufficient specific domain knowledge and experience, using all known methods of interdisciplinary art history and material analysis when necessary. Its validity is initiated by an independent peer review by the best experts in the related artistic field. The connoisseurship practice is based on a bottom-up observation of the artwork immediately followed by top-down categorization and a critical assessment by comparison with other works of the quality and the definition of the final label (attribution). The intrinsic quality of an object concerns its aesthetic properties, the technical skill of the medium (form and beauty) and its originality and uniqueness. However, the intellectual judgment of the connoisseurship asserts that the artistic merit of an object cannot be its intrinsic quality but rather a label attributed by the discerning eye. it is thus the collaborative activity between the sensory areas and the medial frontal cortex that form the basic constituent allowing the access to abstract visual beauty (Rasche et al., 2023).

This intellectual judgment asserts that the artistic merit of an object cannot be its artiness and intrinsic quality but rather a label attributed by the discerning eye. Consequently, the experience of art is subjective, involving cognitive perception, its inferences and bias. The subsequent emotion becomes feeling through the implication of the observer’s personality (Jan De Maere, 2011). The artiness which the observation of an object initiates cannot be evaluated in isolation. It is embedded in its cultural context including social, historical, and political influence (Darda and Chatterjee, 2023), as well as by information and cognitive bias.

Interest in these discoveries should also enhance the field of connoisseurship by providing insights into the neural factors involved in the identification, qualification and evaluation of pictorial works of art.

The future development of scientific studies devoted to neuroaesthetics, a term coined by Zeki (1999) consisting of a scientific approach to the brain mechanisms involved during artistic experience in the widespread sense (Zeki et al., 2020), depends largely on the interaction of at least six fundamental fields of investigation (1) oculomotor skills, (2) vision, (3) oscillatory dynamics of the brain, (4) emotion, (5) memory and (6) identity of the observer. These fields constantly interact, and it is unrealistic to believe that we can understand the mechanisms involved in the perception of art and beauty without considering all these areas of investigation. This review also explores the potential extension of this methodology to the field of expertise and connoisseurship in fine arts to enhance our understanding of the decision-making process involved (Jan De Maere, 2011).

In this inaugural article, we intend to review the current relevance of these three domains to trace a perspective for future practical applications in the neuroaesthetics field ranging from visual search to consciousness.

### 1.1 The artist’s intentions: Vermeer versus bacon

**Figure 1:**
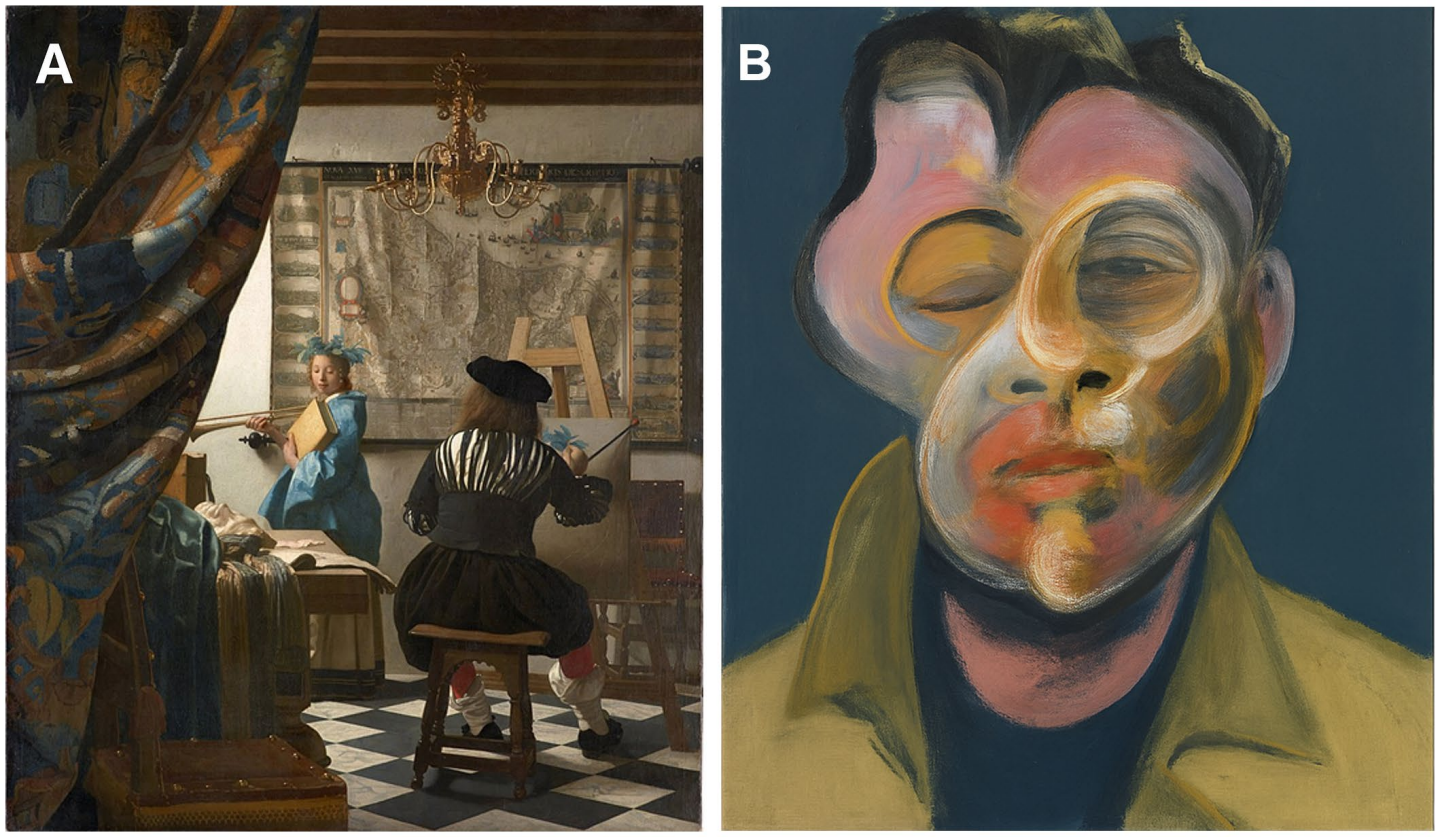
Illustration of Vermeer’s painting “the Atelier” (Reproduced from “Jan Vermeer – The Art of Painting” by Jan Vermeer via Wikimedia Commons under CC0, https://commons.wikimedia.org/wiki/File:Jan_Vermeer_-_The_Art_of_Painting_-_Google_Art_Project.jpg) **(A)** and **(B)** an AI-generated reinterpretation in the visual style of Francis Bacon, Self-portrait, 1969 © The Estate of Francis Bacon, created using OpenAI’s generative tools, used solely for illustrative and academic purposes.

The incommunicability of the artist’s intention is beautifully and symbolically illustrated in Vermeer’s painting “The Atelier” (Figure 1A). In this allegory celebrating pictorial genius, the artist depicts himself from behind, facing his canvas. His gaze is directed obliquely toward his model. To this day, the original message intended by Vermeer remains a mystery, open to various interpretations. Of remarkable expressive merit, this painting ranks among Vermeer’s most technically refined and compositionally innovative works. For the discerning eye, it immediately exemplifies Vermeer’s distinctive quality, individual style, use of materials, pictorial and spatial strategies, and artistic practice. Its apparent simplicity, chromatic vibrancy, formal perfection, compositional innovation, and complex iconographic meaning represent pictorial challenges of the highest order. Vermeer’s composition is functionally intertwined with his iconographic and pictorial strategies, which are seamlessly integrated into the artist’s synthesis and creative vision.

Over centuries, the socio-cultural (art historical) validation by countless discerning observers has established it as a masterpiece, leaving a lasting and profound emotional impact on the receptive viewer (De Maere, 2023).

Bacon’s paintings are a tightrope walk toward a new synthesis of figurative painting following years of its deconstruction. Rather than striving for likeness, the artist declared his goal of delivering an enduring “visual shock” to the viewer by distorting the representation of faces and bodies—categories of stimuli that hold a privileged status in visual perception. Semir Zeki writes: “*Viewing stimuli that depart significantly from the normal representation of faces and bodies entails a significant difference in the pattern of brain activation.*” The face recognition cells of the fusiform gyrus respond more rapidly to caricatures than to normal facial features. The deformed aspects of Bacon’s compositions captivate neurons specialized in facial recognition through their disfluency, presenting a complexity that resists matching with automatic memory prototypes.

The reconstruction of the distorted elements in faces and bodies through emotional color and movement conveys the artist’s inner experiences of suffering, disarray, and desire. There is something archetypal in his primitive touch, evoking a return to the roots of creativity itself. Whether or not this resonates with the observer (the so-called “Intentional Fallacy”), Bacon deconstructs reality to delve into the hidden roots of his subconscious fears and his drive toward destruction. The fragmentation of identity into deformed components avoids mimicry, instead producing basic emotional signs that coalesce into a disturbing yet meaningful whole for many viewers. Bacon paints his tormented identity within a new order, aiming for universality. He situates himself in a black, spaceless box, shifting between mood and persona in a desperate quest for hope, while focusing primarily on painterly quality.

This pictorial embodiment commands the viewer’s attention, leaving them in a state of startling disarray. Facial recognition neurons are unsettled by the ambiguity of the distorted face, struggling to resolve it. Eventually, through top-down cognition, the viewer recognizes it for what it is: a work of art, intended as an illusion that transcends reality.

## 2 The neuroaesthetics complexity

### 2.1 The Shannon’s theory of information is an example of neuroesthetic complexity

An object considered a work of art is part of the broader context of interpersonal communication, spanning from the artist’s intention to the passive and active reception by the observer. This process of communication was first analyzed by Zangemeister and Stark (2007) based on Shannon’s theory of information (Shannon, 1948). According to this framework, the artistic work represents a channel of communication, where the artist is the sender, the viewer is the receiver, and the artwork serves as the message. Within these structural elements, the brain employs various encoding and decoding processes to construct the final perceptual interpretation of the message.

It is important to note, however, that the artist’s intention, as expressed, cannot be received in its entirety by the observer. Instead, the observer projects their own subjective understanding onto the object, recognizing it as a work of art. It is evident that this subjective aspect illustrates the interpersonal differences in appreciation. The ‘Intentional Fallacy’ of the creativity of the artist indicates that the observer freely interpretates its sense and meaning, unhindered by the intention of the artist. The experience of observation of art activates different representations in different viewers at different times.

This illustrates that the experience of art relies on numerous brain processes, encompassing various bottom-up mechanisms, the richness of top-down control exerted by the brain, and their connections to memory, personal experience, and domain-specific knowledge. These complexities can be analyzed using different brain imaging methods, with a precise focus on eye scanpaths that reflect the temporal and spatial exploration of the artwork. This exploration evokes neural signals that range from emotional responses to the final interpretation of the painting.

A striking example of the diversity of artistic intentions and their potential receptions is provided by Francis Bacon’s paintings (e.g., Figure 1B), as studied by Zeki and Ishizu (2013) Bacon’s work demonstrates the direct intention of the painter to evoke a powerful visual and emotional impact.

## 3 Oculomotor skill

### 3.1 The scanpath theory is a strong foundation for the visual search

According to the scan path theory proposed by Noton and Stark (1971), the observation of a painting, an object, or any scene, initiates a series of saccades of the eyes which direct the areas of the retina presenting the highest definition (the fovea) towards certain peremptory elements following a path in the observed object.

The scanpath theory benefits from a unique mode of intermittent control of the visual system called the sampled-data operation (Young and Stark, 1963). This automated process of information sampling is supported by an intermittent movement of the gaze consisting of the production of saccades or micro-saccades reaching a frequency of 4 Hz in the case of reading. The duration of the successive fixations was about 250 ± 50 ms (Henderson and Ferreira, 1993; Krischer and Zangemeister, 2007; Henderson, 2017; Seelig et al., 2021) during which the image of the object is projected into the fovea disposing of a visual field of less than 2° in diameter with the highest cones receptor density allowing color sensitivity and the higher acuity in comparison with the periphery of the retina presenting lower resolution uncolored vision but a high dynamic sensitivity to moving visual stimuli and contrast. In case of viewing pictures the fixation durations (dwell time) are of about 450 ms (Schomaker et al., 2017).

Another issue concerns the exploration of a visual scene, which involves exploratory saccades that move from one point to another along a path (scanpath) precisely defined by the brain. The visual scene appears to us as perfectly unified and flows seamlessly before our eyes. However, the visual cortex receives images only as small areas of high clarity from the fovea—those areas where the saccades have been successively directed along a specific scanpath and followed by position maintenance, allowing the brain to process these visual signals. Beyond these areas projected onto the fovea, the rest of the scene is perceived by the peripheral retina and is therefore blurred. All these fragments contribute to the stabilized ambiguity of the final retinal image of the visual scene.

Retinal persistence should facilitate the task of the brain which must reorganize all the scanpaths carried out over time and reconstruct a visual scene in its relatively clear overall state. Numerous mechanisms, some of which have not yet been discovered, are involved in this function. First of all, the central idea defended by Wurtz’s work lies in the idea that the brain compensates for the jerky, fragmented nature of visual perception produced by eye movements using the principle of corollary discharge (Wurtz, 2018). According to this principle, the brain uses the information stored in memory. This information is then linked to the execution of saccades and the different scan paths carried out (Zangemeister and Stark, 1982a, 1982b; Zangemeister et al., 1995; Brandt and Stark, 1997; Krischer and Zangemeister, 2007).

When the superior colliculus decides to trigger a saccade, this same command is transmitted, via the thalamus (Cavanaugh et al., 2020), to the frontal cortex, where it triggers the reactivation of memory elements and potential automatisms (prefrontal cortex), previously associated with visual perceptions.

**Figure 2:**
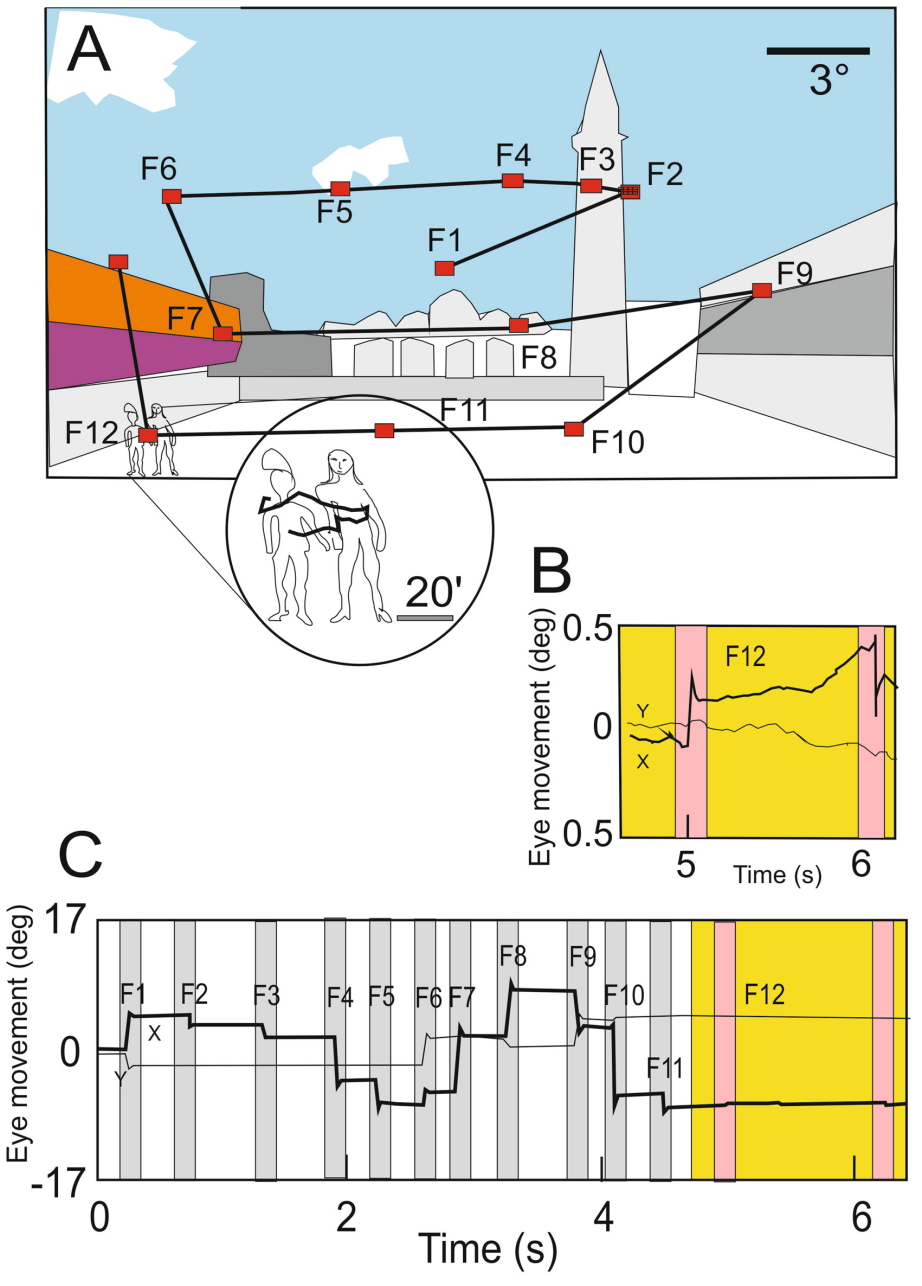
**(A)** Illustration of normal eye movements when an observer explores a scene, a succession of saccades determines the scanpath (yellow lines) separated by short periods of fixation during which visual information is acquired (F1–F13; red dots). **(B)** Horizontal (black line) and vertical (gray line) positions of the oculomotor sequence of the gaze in deg. In the circle, the enlargement shows details of the scene explored by small drifting of the eye during fixation. **(C)** Eye movements (ocular drift) are occasionally interrupted by microsaccades (magenta bars) present during one fixation (F12; circle in panel A and yellow region in B panel), (modified from Rucci and Poletti (2015) with permission).

Between two successive saccades during the fixation period, the eyes are not immobile, incessant microscopic movements are present. The eye wanders following an apparent random drift trajectory occasionally interrupted by micro-saccades (see Figure 2) (Rucci and Poletti, 2015).

**Figure 3:**
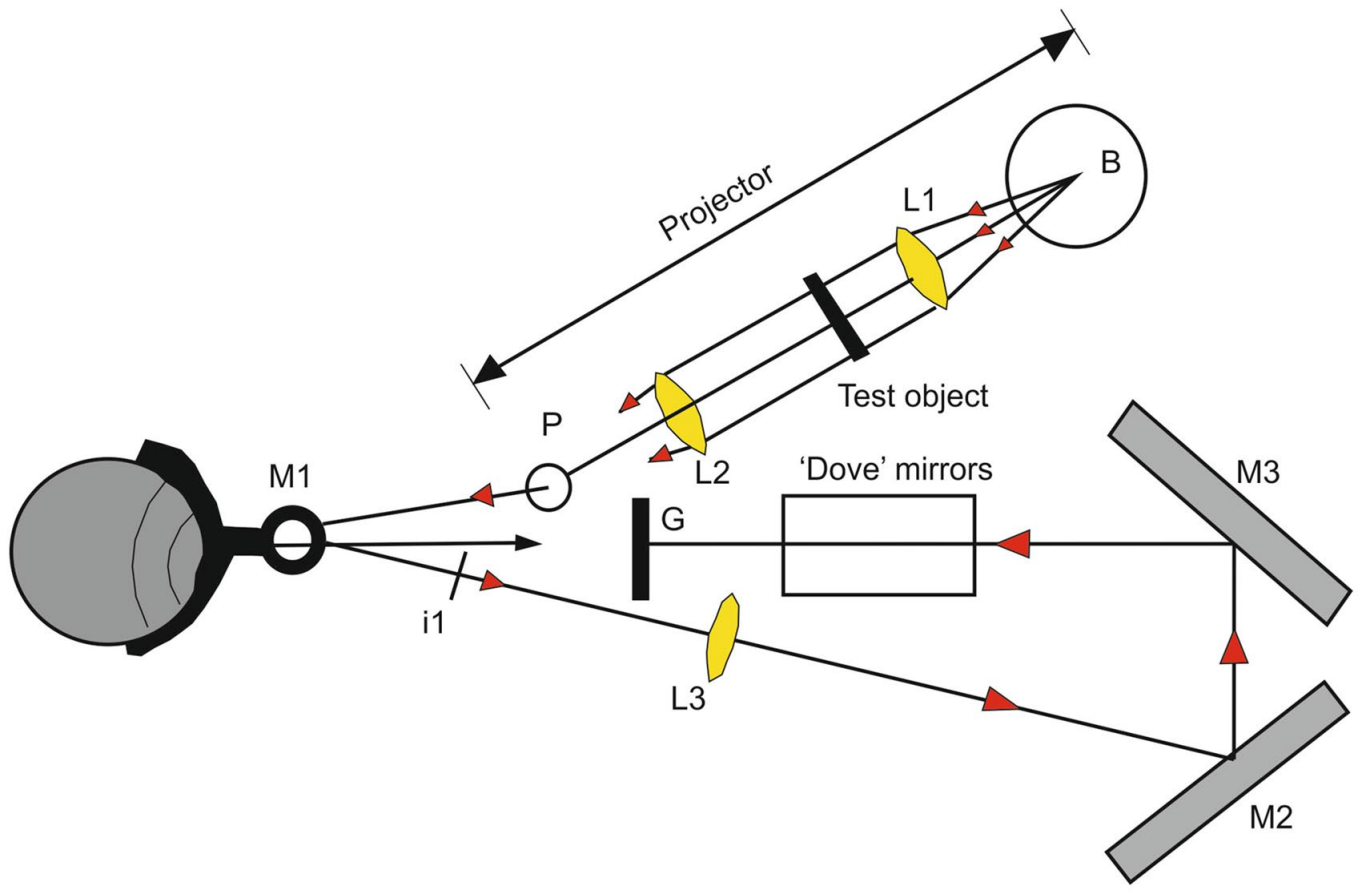
Illustration of the apparatus used for producing a stabilized retinal image. The ‘Pointolite’ lamp (Light of the ball B) is made parallel by the lens L1 and illuminates the object. The lens L2 emphasizes the source on the mirror M1 and forms an image of the object at I1. The lens L3 forms an image of I1 on the ground-glass screen G by reflections at the mirrors M2 and M3, and the subject views this screen directly. The lens L. reverses the direction of motion of the image concerning the eye, but this is corrected horizontally by the mirrors M2 and M3 and vertically by a mirror analog of a Dove prism. The adjustment of the optical system allows that the angular movement of the image on the ground-glass screen to be the same as the angle of rotation of the eyeball. The retinal image is thus stationary concerning the retinal receptors (Modified from Ditchburn and Fender (1955) with permission).

The importance of the presence of these intermittent eye movements during fixation was already demonstrated by the ingenious apparatus of Ditchburn and Fender (1955). This device (illustrated in Figure 3) allows the production of a moving visual target so that its image remains in the same place of the retina despite the presence of involuntary eye movements. This system artificially prolonged the duration of fixation and demonstrates that in this condition the image fades out after about 5 s (Ditchburn et al., 1959).

In addition to the basic refreshing function, fixational eye movements accomplish other functions in visual processing. In this context, it is important to make a clear distinction between saccades and larger micro-saccades participating in scan path exploration of the scene and the fixational eye movements (drift/tremor and smaller micro-saccades) occurring only during fixation periods (Martinez-Conde et al., 2004, 2006).

The fact that human vision is supported by a retina which is never strictly still but agitated by the existence of these fixational movements makes it possible today in the field of neuromorphic vision (Douglas et al., 1995) to imagine new processes for capturing images integrating these fixational movements (Testa et al., 2023). Neuromorphic vision concerns processing inspired by the structure and function of biological vision systems. It attempts to mimic how biological systems process visual information. For this aim, even-based cameras detect changes in light intensity at individual pixels.

### 3.2 Saccadic and blink visual suppression

If saccades are essential to enable efficient foveal vision in a complex environment, as we have seen previously, they make the functioning of the visual system more complex. It is well-recognized that we are blind during the movement phase of the saccade (Zuber and Stark, 1966). This suppression of visual input is unconscious. Our impression of a continuous visual perception as not being interrupted by saccades and blinks is firstly due to the retinal persistence (Coltheart, 1980) supported by the feedforward-feedback laminar network in the retina (Coltheart, 1980; Yeonan-Kim and Francis, 2019). This persistence is of the order of 50 ms after the switch-off of the image on the retina.

Saccadic suppression is an important phenomenon for visual perception; it occurs before the saccade is triggered, which raises the possibility that an active mechanism is at work. How can we explain that many neurons in the visual cortex are inhibited 80 ms before the start of the saccade and 55 ms before the triggering of a blink? As explained before, the dominant idea is that there is a corollary discharge (Wurtz, 2018) coming from subcortical and cortical regions involved in the programming and triggering of saccades or blinks. Given that this motor programming will cause a certain delay before the execution of the saccade, a corollary discharge should be able to inform the neurons of the visual cortex of the arrival of the saccade and initiate the suppression mechanism.

The theory of scanpath associated with the analysis of other gaze strategies (pursuit and head-eye coordination) (Levin et al., 1982; Stark, 1983) has also paved the way for the study of eye movements in psychiatry (Pereira et al., 2014, 2020; Wolf et al., 2023). This demonstrates the functional intricacy of the genesis and control of eye movements within cognitive cerebral functioning. These movements indeed present stereotyped alterations both in schizophrenia (Levy et al., 2010; Wolf et al., 2021) and in Alzheimer’s disease (Wolf et al., 2023).

It is interesting to know that using a forced-choice saccade task presenting two different images on the right and left side, Kirchner and Thorpe (2006) demonstrated that human subjects can produce a saccade at a short latency of 120 ms towards the target image. This means that the visual processing along the ventral stream is very fast for the programming of the saccade. In the same context, It was recently demonstrated (Castellotti et al., 2023) that fragmentary images presented for a duration of 25 ms can be rapidly discriminated when they contain optimal local information providing salient features necessary for rapid visual feature extraction (Castellotti et al., 2021). A model based on constrained maximum entropy presented by the same group allows the optimization and the speed-up of visual image reconstruction (Del Viva et al., 2013).

**Figure 4:**
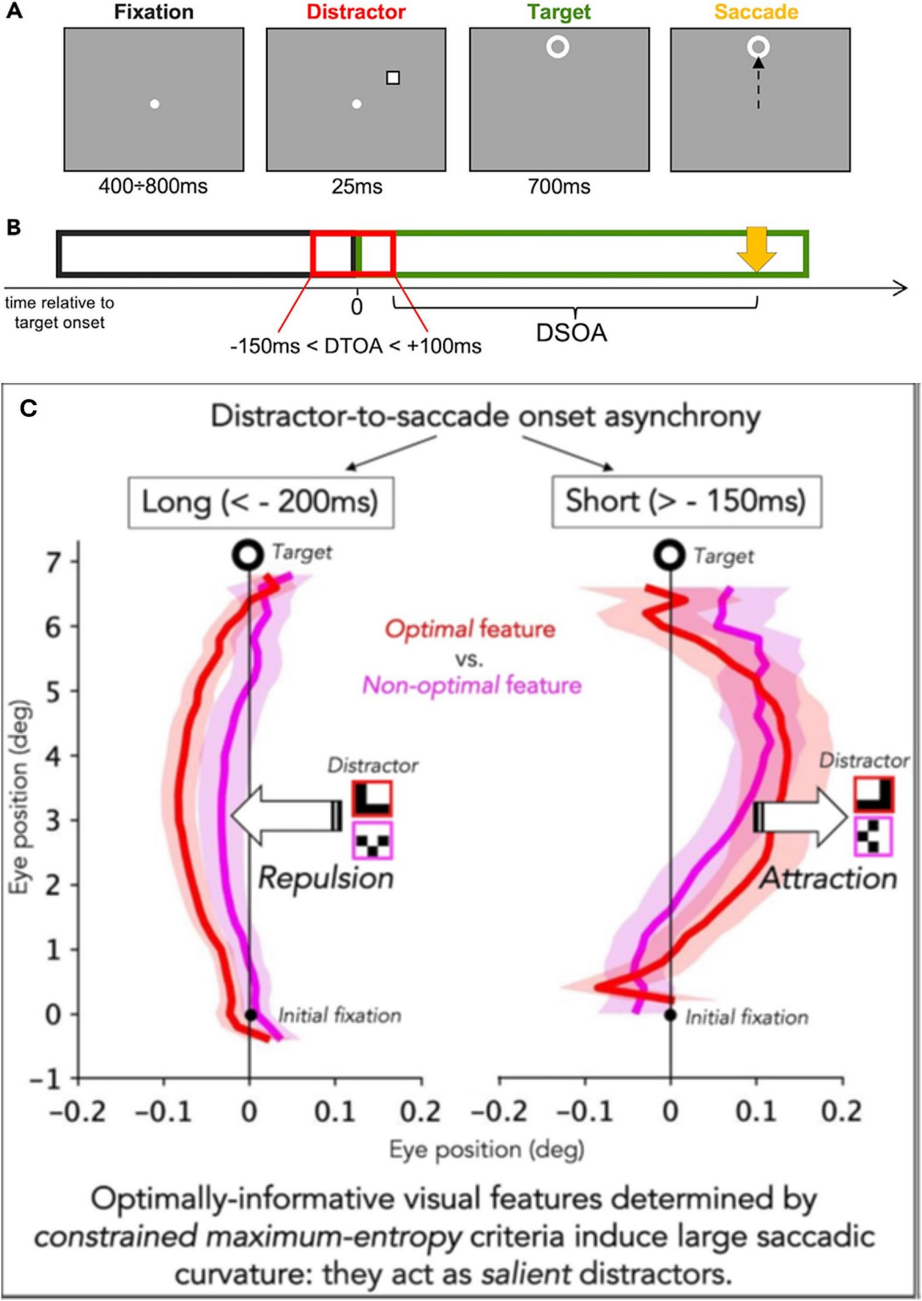
Illustration of the experimental condition. **(A)** Example of a trial. The duration of the different stimuli is reported under each panel. In this example, the distractor is presented as an empty square and had a negative distractor to target onset asynchrony (DTOA) (presented before the target), and the target is presented 7 deg above the fixation. **(B)** Display sequence. The fixation point disappears as soon as the target appears. The time 0 refers to the target onset. Relative to target onset, the distractor could appear from 150 ms before the target to 100 ms after the target. **(C)** The time distance between the distractor offset and the saccade onset (yellow arrow) is the distractor offset-to-saccade onset asynchrony (DSOA). When the DTOA is < −200 ms the saccade is deviated away from the distractor (repulsion); oppositely, when the DTOA is > −150 ms the saccade is deviated toward the distractor (adapted from Castellotti et al. (2023), CC BY 4.0).

This model is founded on the need for a strong data reduction that must be operated by the visual system at an early stage, to optimize and speed up the reconstruction of visual images. In the same vein, the presentation of salient distractors just before the onset of a saccade directed to a target can change the curvature of the saccade. The magnitude of the curvature change depends on the optimal visual features of the distractors (Figure 4). The direction of the curvature depends on the presentation time of the distractor concerning the time presentation of the target. If the distractor arrives before −200 ms the increased curvature is directed toward the opposite side of the distractor (repulsion). Inversely if the distractor arrives after −150 ms the increased curvature is directed toward the distractor (attraction) (Castellotti et al., 2023). This experiment confirmed that the visuo-oculomotor system can rapidly process the presence of optimal visual features and generate a dynamic attraction or repulsion curvature of the saccade trajectory.

### 3.3 The fixational eye movements and sensory-motor mnemonic trace

Although traditionally regarded as a simple mechanism to refresh neural activity and prevent perceptual fading, a growing body of evidence indicates that fixational eye movements play a more central role in vision. These movements are now known to modulate neural responses in various cortical areas, such as MT (Hohl and Lisberger, 2011). Multiple studies support the idea that they are involved not only in the acquisition of visual information but also in its processing (Martinez-Conde et al., 2004, 2006; Costela et al., 2017). For example, recent research demonstrated that the disappearance of stationary images (Troxler fading), as exemplified by the sun in Monet’s painting, was more pronounced when the rate of micro-saccades was reduced (Alexander et al., 2021). The authors concluded that oculomotor dynamics during fixation contribute to a cornerstone of Impressionism. The aesthetic impact of this painting is further enhanced by Monet’s intentional use of “iso-luminance” (Jan De Maere, 2011).

**Figure 5:**
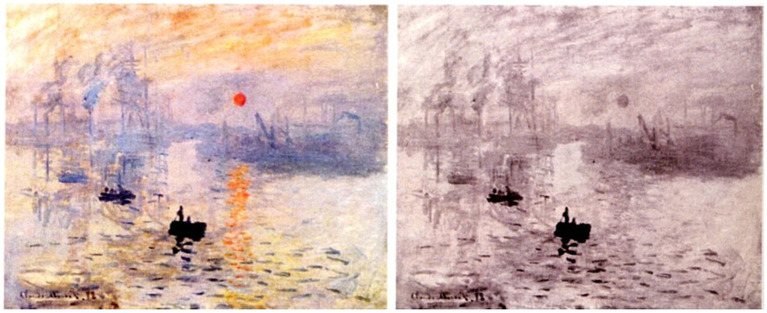
Illustration of Claude Monet, “Impression du Soleil levant”, Musée du havre (Fr). Reproduced from De Maere, J. Neurosciences et connoisseurship: la physiologie du Beau et l’attribution des Tableaux Anciens, https://lib.ugent.be/en/catalog/rug01:002963364.

Although traditionally regarded as a simple means to refresh neural activity and prevent perceptual fading, a plethora of mounting evidence indicates that fixational eye movements play a more central role in vision. These movements are now known to modulate neural responses in various cortical areas such as in MT. Multiple studies support the notion that they are involved not only in the acquisition of visual information but also in its processing. For example, it was recently demonstrated that the disappearance of stationary images (Troxler fading) in the case of the sun in Monet’s painting (Figure 5) was more intense when the micro-saccade rate was reduced. These authors concluded that the oculomotor dynamic during fixation contributes to the cornerstone of Impressionism. The aesthetic impact of this painting is greatly enhanced by Monet’s incident potential of “iso-luminance” (Jan De Maere, 2011).

The successive fixations and the trajectory followed by the movement of the eyes are most often characteristic of the painting or the scene and are reproduced by learned automatisms (prefrontal cortex) when the same object is again presented to the subject.

The scanpath makes it possible to acquire a sequence of visual information sampled over time which represents a visual and cognitive memory. The exploratory motor sequence itself constitutes a sensory-motor mnemonic trace of what has been perceived. It is in fact on these two fundamental aspects of the scan path theory that other neuroscientific theories will be grafted, involving the complex processes of visual physiology and of bottom-up and top-down dialogues.

Numerous pieces of evidence supported the idea that the scan path movements of the eyes are linked to mnemonic processes (Brockmole et al., 2006; Henderson, 2017). Because visual perception is time-bound, the temporally discontinuous inputs obtained by successive scanpath-induced fixations must be linked to the working memory [visual short-term memory (VSTM)]. It is a question of integrating over time the different spatially isolated percepts according to the order prescribed by the ocular exploration (Brockmole and Irwin, 2005).

However, it remains discussed if the scan path movements are a sensory-motor mnemonic trace working in parallel independently to the visual processes, or in a complementary manner. This latter possibility is supported by the fact that repeated presentation of the same object elicits fewer fixations and sampling regions (energy saving automatisms) as if previous presentations had stored data that need not be secure again (Ryan et al., 2007).

In the same vein, repeated presentations increased the accuracy of target memory locations (Althoff and Cohen, 1999; Stacey et al., 2005), the speed of target search (Chau et al., 2011), the detection speed of invariant targets in comparison to novel ones (Chun and Jiang, 2003; Brockmole and Henderson, 2006).

The relationship between eye movement and mnemonic content relating to observed objects can be better understood by referring to the reading of a text. In this case, to read English the gaze moves mainly from left to right. Most words are read and recognized, but some are ignored and others are reread several times, which involves looking back so that previously read text can be reread (Henderson and Ferreira, 1993).

This predictive capacity would mean that the understanding of the text being read should be directly available so that the ocular motricity can trigger the next forward saccade in search of the new word containing the most appropriate semantic and syntactic information for understanding the final content of the message.

This reflection from the field of reading can be applied to the exploration of a painting which, like a text, can be managed in a predictive way by the scan path of the gaze directing itself according to certain more striking or significant pictorial elements (pop-out) (Peacock et al., 2023) than others and linked together by the emotional and cognitive context carried by top-down influxes. In this situation, the return of the gaze to elements already explored can indicate the interest and the questioning brought by these pictorial elements to the understanding of the work.

In an Opinion Paper, Henderson (2017) posed a series of outstanding questions that may pave the way for future scientific explorations of the artistic domain:

1. How precise are the spatial predictions that are used to guide gaze?
2. Are the predictions that guide gaze used strategically or automatically?
3. Can the prediction approach to gaze control in natural scenes be extended to gaze control in tasks such as face perception, reading, and spatial navigation?
4. Can the prediction approach to gaze control be extended to account for both where fixation is directed and how long fixation is directed there?
5. How are the memory structures that generate predictions for gaze control encoded, stored, retrieved, and deployed?

### 3.4 Meaning maps versus saliency maps

**Figure 6:**
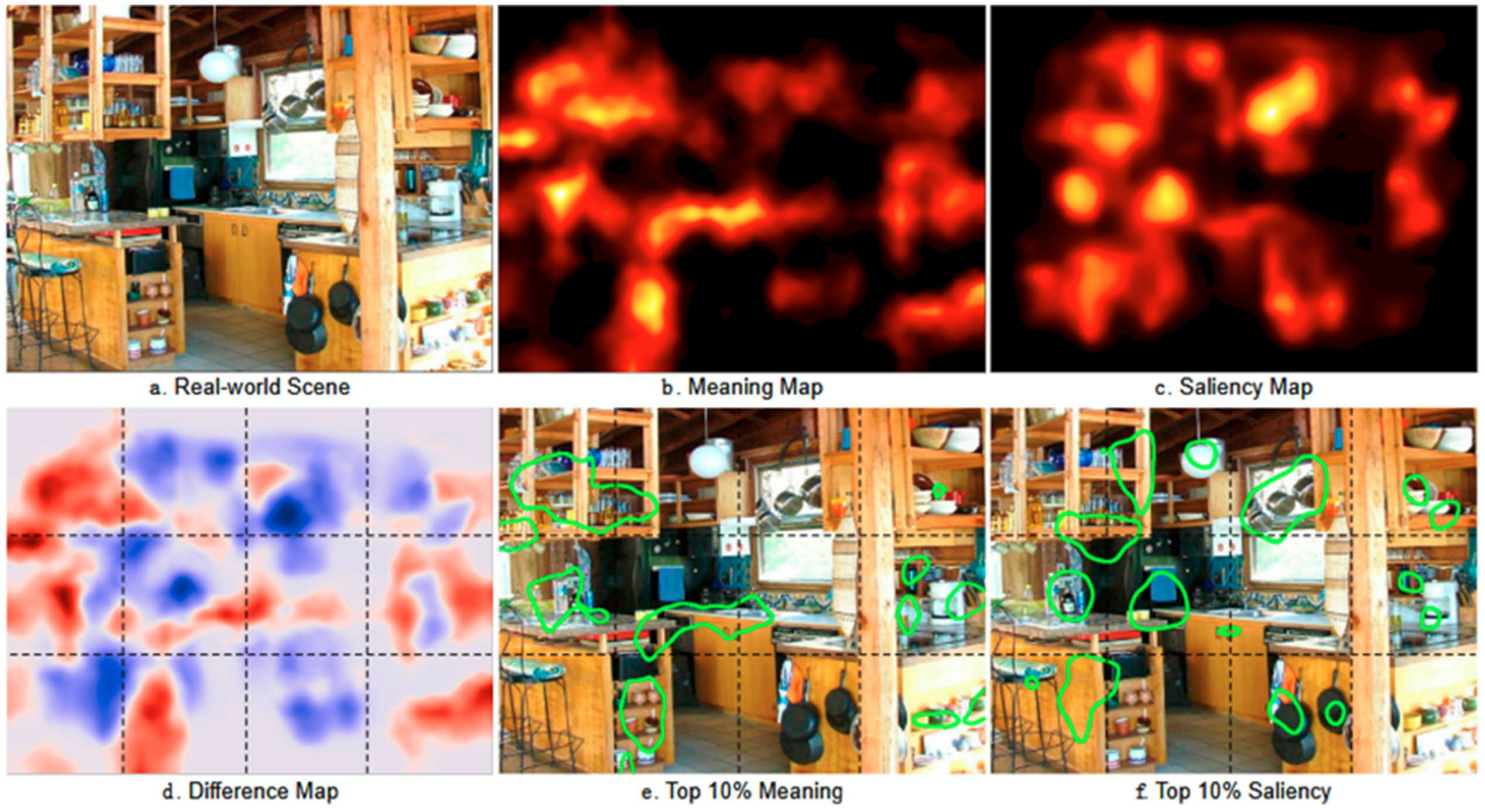
Meaning and saliency maps for a real-world scene. In **(a)**, the real-world scene on which the eye-scanpaths are performed to extract the meaning map **(b)** and the saliency map **(c)**. In **(d)**, the difference between the meaning and the saliency maps. In **(e)** and **(f)**, the top 10% of the meaning and the saliency maps are highlighted, respectively (reproduced from Henderson et al. (2019), CC BY 4.0).

The concept of meaning maps, introduced by Henderson and Hayes (2017) captures the spatial distribution of semantic information in real-world images. Semantic information refers to the meaningful content or interpretation of elements in an image. This includes the identification, categorization, and understanding of objects, actions, and relationships present within the visual scene. Similarly, saliency maps, as proposed by Itti and Koch (2001) and Elazary and Itti (2008), encode the spatial distribution of luminance contrast, edge orientation, and color (see Figure 6). Attentional guidance can thus be influenced by saliency maps primarily driven by bottom-up mechanisms (stimulus-driven attention) and/or by meaning maps supported by top-down mechanisms, including cognitive memory.

To construct meaning maps, visual scenes are systematically divided into small parts (patches), which are then presented individually to multiple participants for evaluation of their meaningfulness and assignment of a score. This score is represented as a colored zone overlaid on the original scene, facilitating comparison with the saliency map. Previous research has shown a high correlation between meaning and saliency (Tatler et al., 2011) prompting the empirical question of which map ultimately guides attention.

Eye movement recordings during scene observation were used to address this question, comparing the distribution of fixations over time with meaning or saliency maps (Henderson and Hayes, 2017). While it might be intuitive to suggest that the saliency map, driven by direct input from the retina, operates first in the attentional process, followed by the slower influence of the meaning map (Anderson et al., 2015). Henderson and Hayes (2017) demonstrated that the meaning map is a superior predictor regardless of timing. The prevalence of the meaning map cannot be explained solely by central bias, the tendency for viewers to fixate on scene centers (Peacock et al., 2020).

In a recent study, the same group concluded that the meaning of an object plays a crucial role in selective attention during passive scene viewing (Peacock et al., 2023).

### 3.5 Integrated priority map and working memory

While top-down and bottom-up processes are often defined as distinct, it’s important to recognize that they can interact, giving rise to a third process where past attentional selection, based on history, integrates with current goals and physical salience. All together these elements form an integrated priority map (Awh et al., 2012) in which the working memory (WM) plays an important function (Baddeley, 2003; Hamamé et al., 2012).

Working memory refers to our ability to hold a small amount of information for ongoing cognitive processes, with a limited capacity compared to episodic long-term memory (LTM) (Cowan, 2001). The content of the WM is protected against proactive interference (PI) coming from the information contained in the LTM (Oberauer and Awh, 2022; Oberauer and Greve, 2022). It was concluded that the information flow was flexibly gated from the LTM to the WM only when this transfer was helpful (Mızrak and Oberauer, 2022).

Recent research has revealed that working memory, with its recurrent connections, can sustain attentional demands (Lansner et al., 2023), allowing information to be retained despite temporary interruptions in working memory activity, a phenomenon termed “silent” working memory (Wolff et al., 2017; Oberauer and Greve, 2022).

However, the neurophysiological mechanisms underlying WM are complex and subject to debate (see, for example, Barbosa et al., 2021; Fields, 2022). Consequently, new studies that integrate eye scan paths and EEG dynamics during visual exploration are highly valuable.

### 3.6 Scanpath exploration and consciousness

About the ever-increasing complexity of knowledge concerning both structural and functional aspects of the circuitry involved in visual search, which remains essential to better understand the working mechanism of connoisseurship expertise, the question of the emergence of consciousness in decision-making appears as an essential element.

Based on neuroanatomical data and experiments about the perception of color and motion, Zeki suggested that visual consciousness is not a single unified entity, but consists of many micro-consciousnesses occurring at different times and in different nodes in the neuronal space (Zeki, 2001, 2015). He writes: *the consequence of spatially and temporally distributed micro-consciousnesses is that their integration is a multistage, nonhierarchical process that may involve a neural “glue.”* This theoretical position of post-binding is different from the most popular idea that it is the binding itself that generates consciousness (Crick and Koch, 1990; Engel and Singer, 2001; Fries et al., 2001).

The decision of a connoisseurship expert also involves the activation of the self-awareness network, which plays a crucial role in conscious experiences, particularly in self-monitoring and behavioral control (Lou et al., 2017). This network is in the medial parietal cortex, the posterior cingulate cortex, as well as the medial prefrontal cortex and the anterior cingulate cortex. It is composed of numerous neuronal “hubs” interconnected by gamma oscillations (40 Hz) generated by GABAergic inhibitory interneurons and regulated by dopamine. This network also requires a high metabolic rate, supported by an adequate oxygen supply (Lou et al., 2020).

## 4 The framework of visual processing

### 4.1 The basic visual circuits and the major specialized visual pathways

After the first treatment of the visual input by the retina, the neural signal is conveyed by the axons of the magno- and parvocellular ganglion cell (each eye containing 1 million optics nerve axons) reaching the V1 visual cortex through the lateral geniculate nucleus (LGN).

Several studies demonstrated that form and motion information are treated separately by ventral and dorsal visual streams, and converge to the posterior portion of the superior temporal sulcus (Vaina et al., 2001; Giese and Poggio, 2003; Blake and Shiffrar, 2007) (Figure 6).

In addition to this classical subdivision between the ventral stream named a ‘*What*’ pathway supportive of object identity and a dorsal stream called a ‘*Where*’ pathway supporting the location and motion related to the object, the dorsal stream give rise to three sub-pathways dedicating to (1) visually-guided actions (premotor cortex), (2) spatial working memory (medial temporal lobe) and (3) navigation (retro-splenial areas) (Kravitz et al., 2011). More recently, a third visual pathway dedicated to social perception (Pitcher and Ungerleider, 2021) projects from V1, via V5/MT areas, into the superior temporal sulcus (STS). This third pathway is representative of face and body moving expression including diverse dynamical aspects of social perception, from face and gaze expression, and audio-visual integration to intention and mood (Pitcher and Ungerleider, 2021).

### 4.2 The superior colliculus and the corollary discharge

From V1, the visual input is also dispatched to the posterior parietal cortex (in particular the lateral intraparietal region, LIP) and the dorsal frontal cortex (in particular the frontal eye field, FEF). From these cortical regions, the visual information is sent back to the superior colliculus (SC) which is the timetable structure to program the saccade (Sparks, 1986) finally performed by the midbrain and brainstem circuits (Robinson and Keller, 1972; Cheron et al., 1986; Godaux and Cheron, 1996) up to the motoneuronal pool and the extraocular muscles. The SC is a layered entity occupying a crucial crossroad in the control of saccadic eye movement. The SC is implicated in the sensorimotor transformation consisting of the process of first sensing an element in the environment for producing a saccade in response to that element (Ayar et al., 2023).

To produce a voluntary ocular saccade, a higher-order command of cortical origin (frontal cortex) informs SC of the interest in triggering a saccade towards a visual target present in the environment. The SC is a complex structure composed of 3 layers of neurons, a superficial layer which is a true internalized retina, an intermediate sensorimotor layer, and a deeper layer of motor neurons (Sommer and Wurtz, 2002, 2008). It is these latter neurons that will trigger the saccade by activating the saccade-generating neurons located in the reticular formation of the brainstem and the midbrain. These latter neurons produce bursts of high-frequency action potentials proportional to the speed of the saccade. Once activated, these burst neurons activate the motoneurons and the saccade is produced.

The SC motor map is interconnected with the parietal and frontal cortex, with the basal ganglia, and with the premotor circuitry (Sparks, 1986; Gandhi and Katnani, 2011; Krauzlis et al., 2013). It plays also a central position in eye-head coordination (van Opstal, 2023). This explains that electrical stimulation in one point of the SC retinotopic map caused the eyes to move toward that point of the visual field which projected to that point of the SC (Robinson, 1972).

The recent study of Yu et al. (2024) demonstrates that SC visual neurons can preferentially respond to face within 50 ms of stimulus onset. Thus, largely in advance to the face recognition exerted by the in visual cortex. This short-latency face preference is explained by a direct connection between the LGN and the SC and may thus contribute to the preparation of voluntary saccadic scan path as such performed by a connoisseurship which could be automatically directed to face element well before the recognition of individual faces by the higher-order visual area. This also confirms the implication of the SC neurons in the endogenous attention phenomenon (Ignashchenkova et al., 2004; Cavanaugh et al., 2006) and occupies a strategic position in the oculomotor theory of attention proposed by Rizzolatti et al. (1987).

Following this theory, our attention is directed to a given point of the visual field when the oculomotor program for moving the gaze to this point is ready to be executed. The corollary discharge emerging from the SC may play this role in attention and visual search mechanism (Wurtz, 2018).

It is interesting to note that V1 collects not only retinal information but also important recurrent influx from other cortical zones, the modulatory input from the thalamus, and projections from the brainstem. In addition, nonretinal projections represent active modulation conveying specific information (Singer, 2021; Zatka-Haas et al., 2021; Pinto et al., 2022). It was recently demonstrated that the same V1 neuronal population can simultaneously treat information about a visual stimulus and the related forthcoming choice before the onset of the motor response (Yiling et al., 2023a; Yiling et al., 2023b).

However, it is not proven that this latter neuronal activity linked to the selection of the appropriate response is carried out locally within V1. Rather, this V1 activity is most likely induced by top-down inputs from other cortical regions. The existence of a top-down entry explains not only the delay between the activity of recognizing the object and the activity linked to the choice of the response but also the clear separation between these two periods of activity. Current data (Yiling et al., 2023a; Yiling et al., 2023b) suggests the existence of subspaces within V1 associating dynamics linked to local recurrent loops involving the different interneurons and pyramidal cells of the different layers and top-down loops originating from higher-order cortical areas including corollary discharges motor commands. The abundance of reciprocal recurrent loops is the basis of complex dynamical landscapes working in parallel that can switch over time into relatively independent subspaces to avoid interference between the different functions (visual recognition versus behavioral decision). In this context, the presence of traveling waves (Davis et al., 2021; Benigno et al., 2023) (see below) is one of the numerous signatures of this complex dynamics. Recently, it was demonstrated that this complex dynamics included the brainstem which is hierarchically organized into integrative hubs of nuclei presenting similar cortical connectivity implicated in numerous functions (Hansen et al., 2024).

### 4.3 The mirror neuron network

Initially discovered in the F5 area and inferior parietal lobe (IPL) of the macaque (di Pellegrino et al., 1992), the mirror neurons are a particular class of visuomotor cells. Their specificity lies in discharging both when the monkey performs an action (for example, “grasping an object”) and when, at rest, it observes a fellow, whether monkey or human, performing the same action. Thus, these neurons are not directly concerned with the exploration of a passive pictural image but are well-engaged during the observation of any type of action performed during artistic gesture (Zarka et al., 2022). In addition, the observation of static images depicting dynamics action can activate the MMN (Proverbio et al., 2009).

During execution as well as observation of a gesture, their response begins at the start of the movement and stops at the end, and is therefore not linked to motor task preparation (Gallese et al., 1996).

Moreover, mirror neurons in monkeys are only stimulated by actions, meaning gestures with a certain semantic value (transitive or intransitive). They do not activate mimed gestures, meaningless gestures, or emotional gestures. The motor theory of perception, is based on the fact that movement perception is influenced by implicit knowledge about the working principles of motor control (Rizzolatti and Craighero, 2004).

**Figure 7:**
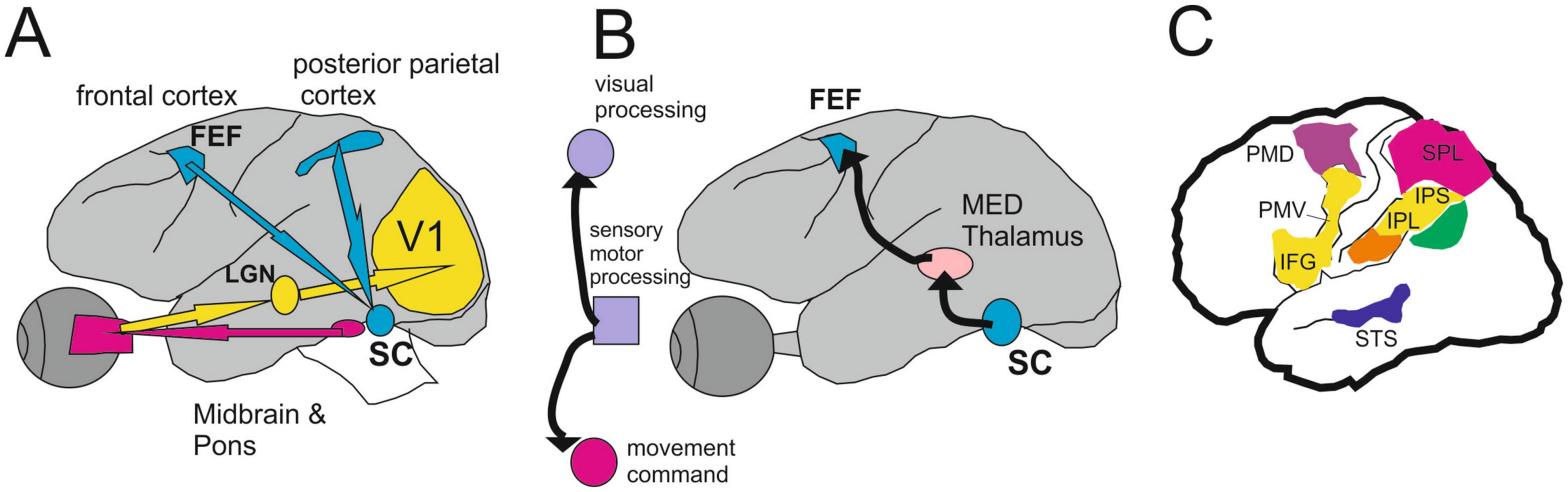
The circuit from the retina to the mirror neurons network. Monkey Brain circuits for visually guided saccades **(A)** and saccade-based corollary discharge **(**CD, **B)** (Modified from Wurtz, 2018). See text for more details. **(C)** Cortical areas of the human brain are related to the parietofrontal mirror system responding to different types of motor acts. Yellow indicates transitive distal movements; purple, reaching movements; orange, tool use; green, intransitive movements; blue, portion of the superior temporal sulcus (STS) responding to observation of upper-limb movements. IFG indicates inferior frontal gyrus; IPL, inferior parietal lobule; IPS, intraparietal sulcus; PMD, dorsal premotor cortex; PMV, ventral premotor cortex; and SPL, superior parietal lobule (Modified from Cattaneo and Rizzolatti (2009)).

In this context, it is useful to outline the basic circuitry from the retina to eye muscles to demonstrate the large involvement of different cortical and subcortical long-range loops which are constantly present during any type of experiments involving visual search. It is well demonstrated that a mirror neuron network (MNN) exists in humans. The observation of motor acts produces a modulation of the EEG mu rhythm analogous to that occurring during motor act execution. In particular, the cortical motor system closely follows the velocity of the observed movements (Avanzini et al., 2012). In addition, Koch et al. (2010) demonstrated that the anterior intraparietal cortex (AIP) and the ventral premotor cortex (PMv) were activated when the subjects observed goal-directed grasping performed by another subject. The following regions including the inferior frontal gyrus (IFG), the inferior parietal lobule (IPL) including the angular gyrus, and the posterior part of the superior temporal sulcus (pSTS) participate actively in the MMN (Rizzolatti and Craighero, 2004; Cebolla et al., 2014) (see Figure 7).

## 5 Oscillatory dynamics of the brain

### 5.1 Spatial attention and alpha oscillation

Spatial attention occupies a central role when looking at a painting, it enhances the speed and quality of the scan path directed toward targeted locations (Carrasco, 2011). To better understand the complexity and various intricacies revealed by numerous psychological studies, it is increasingly useful to address the neurophysiological correlations of human attention. It is well-admitted that selective attention results from two major mechanisms: target selection and distractor suppression (Foxe and Snyder, 2011). However, the independence of these mechanisms remains highly debated (Wöstmann et al., 2019; Schneider et al., 2022).

The first dominant oscillatory activity in humans in the resting state, the alpha rhythm (8–12 Hz) is the one appearing to play an important role in the processes of visual attention. It was mentioned that the topographic distribution of alpha power is reduced (ERD) at the scalp contralateral to an attended location (Kelly et al., 2006). During the same experiments these authors showed an increase of the alpha power over the ipsilateral (ignoring) hemisphere suggesting that alpha power increase (ERS) corresponds to a mechanism of active attentional suppression of the informational content present in the non-attended visual hemisphere. This physiological interpretation is by the inhibition-timing hypothesis of the alpha rhythm defended by Klimesch et al. (2007).

This suggests that measuring the power of the alpha which accompanies the exploratory saccades of a common subject, or an expert could tell us what pictorial information is subject to inhibition and what are its cognitive, cultural, and emotional attributes, at the basis of this attentional suppression. The tracking of the alpha power of an observer toward spatial attended or not attended location has been already performed concluding that these spatially alpha power signature provides the direction of spatial attention (Popov et al., 2019) and may also predict the future spot of selective attention (Hanslmayr et al., 2011; Saurels et al., 2021).

In the same vein, the phase of the ongoing EEG oscillations is another mechanism that may help to make distinction between the different functions played by these oscillations (Harris et al., 2018). These authors demonstrated that the detection of unattended targets was more related to the 5 Hz theta phase oscillation than the detection of attended targets. In contrast, the phase of the alpha oscillation was not related to this attentional role but well to the perceptual ability.

The alpha oscillations are involved in at least 5 functions or characters: (1) global resting state, stabilizer; (2) selective attention, perceiver; (3) cognitive performance, predictor; (4) inhibition and gating, inhibitor; (5) consolidation of new motor sequence (sleep-spindle) (Cheron et al., 2016; Clayton et al., 2018). By using neural representational dissimilarity matrices on MEG recordings, Kaiser (2022) demonstrated that alpha and beta oscillations track our aesthetic judgments of natural videos allowing the possibility to decode the brain signature of beauty and a separate one for art.

### 5.2 Travelling waves

In direct relation to this last point, the existence of mobile oscillation (traveling wave, TW) may help us to decipher the subtle mechanism implicated in neuroaesthetics. TW occurs during different brain states ranging from low-frequency oscillation in sleep to higher frequency oscillation in awake situation. TW may perform various functions such as those involved in dynamic processing of sensory information to memory consolidation and recall (Muller et al., 2018). TW were studied in the visual cortex of the monkey (Davis et al., 2021) and treated with recurrent neuronal models (Benigno et al., 2023). This group was able to explain how the networks of the visual cortex could predict with high accuracy complex and naturalistic inputs.

Recently, Zabeh et al. (2023) demonstrated in monkeys that TWs in the beta band circulating between the frontal and parietal cortex were more intense when eye saccades were directed towards targets previously associated with reward than those directed towards neutral targets. The phase delays between TW oscillations between different brain regions and the thalamus may also provide useful paradigms to understand the mechanism of the temporal binding (Alamia and VanRullen, 2019, 2024). The same group demonstrated that when attention is allocated to one visual hemifield the alpha TW propagates from frontal to occipital areas ipsilateral to the attended location. Conversely, alpha TW propagates from occipital to frontal areas contralateral to the attention side (Alamia et al., 2023).

### 5.3 Gamma wave in visual suppression

Gamma oscillation (30–100 Hz) occupies a central position in cognitive neuroscience. The contemporary understanding of gamma oscillation points to its emergence from the synchronous activity of a large ensemble of firing neurons (Eckhorn et al., 1988; Gray et al., 1989; Jensen and Colgin, 2007). It is central to the binding theory, in which gamma oscillations combine different features in a visual scene to form a coherent percept (Singer, 1999). For example, the observation of video of walking human avatar in upside-down condition elicited gamma power increase at about 150 and 400 ms and a gamma ERD at the same latency in uncoordinated condition (Zarka et al., 2014). The subject was not instructed to perform any mental task, but implicit recognition can recruit gamma activity for unconscious and conscious neuronal process (Aru et al., 2012; Vidal et al., 2014). These results are in agreement with the data of Tallon-Baudry et al. (1996) where they demonstrated the presence of non-phase-locked gamma activation (60 Hz) at about 300–400 ms after the presentation of an illusory Kanizsa triangle figure.

The complex interplay of these gamma wave occupies a central position in cognitive neuroscience (Kandel, 2013). In the context of the Global Workspace Theory, serial and parallel processing take part from the widespread treatment of unconscious information to the emergence of consciousness (Baars, 1988; Changeux and Dehaene, 1989; Mashour et al., 2020).

A recent study in macaque (Denagamage et al., 2023) demonstrates the existence of the corollary discharge at the level of the high-order V4 zone activating the inhibitory interneurons of layer IV for producing the visual suppression well before the saccade onset. Layer IV of V4 receives the visual information from the lower-order zones V1, V2, and V3 (Ungerleider et al., 2008). At the same time, the superficial (II/III) and deep layer (V/VI) of V4 receives input from the prefrontal cortex (FEF) and subcortical structure as the pulvinar. These latter inputs convey the corollary discharge related to the saccade programming. These influx were then treated by V4 circuitry and then transmitted to downstream visual areas, such as the superior temporal cortex (ST) (Boussaoud et al., 1990), the inferior temporal cortex (TEO) (Distler et al., 1993) and the visual association area (TE) (Borra et al., 2010).

**Figure 8:**
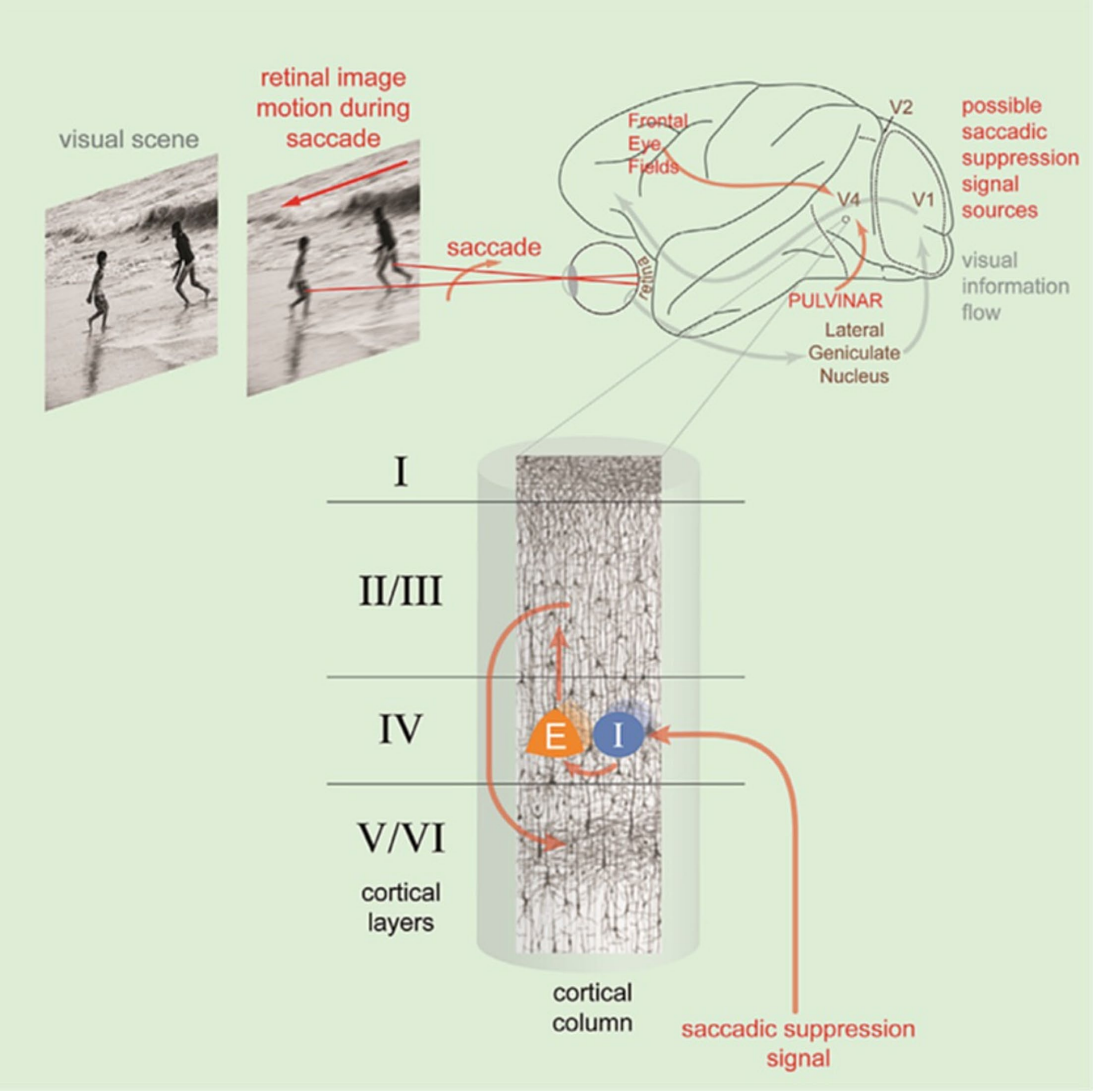
Illustration of the saccadic suppression linked to the retinal image motion occurring during saccade. This is mediated by a saccadic suppression signal input to layer IV of area V4 activating the interneuron (I) of layer IV which inhibit the pyramidal cells (E) of the same layer. These excitatory cells project to layer II/III and from there to layers V/VI. The saccadic suppression signal comes from the Frontal Eye Fields and the pulvinar (reproduced from Denagamage et al. (2023), CC BY NC ND).

The contribution of cerebral oscillations to the mechanisms of visual suppression accompanying saccades and blinks was suggested by different groups (Lachaux et al., 2008; Ramot et al., 2012; Kuzovkin et al., 2020). According to these studies carried out using intracerebral recordings, rapid gamma-type oscillations (20–150 Hz) can play an important role either in a positive way as a synchronizing acceleration (‘*accélération synchronisatrice’*) anticipatory named by Bremer et al. (1960), cited by Steriade (2000). In agreement with the oscillatory theory of Singer (Gray et al., 1989; Singer, 2013), the gamma wave is now recognized to participate in the active processes of visual perception and also in a negative way through a disruptive desynchronization playing an active role in visual suppression as recently reported by Denagamage et al. (2023) (Figure 8).

These authors demonstrated that saccadic neural suppression in V4 points around 125 ms after saccade onset. It is important to note that this neuronal suppression must occur when the visual information linked to the image motion on the retina during the saccade arrives in V4. It is therefore necessary to consider the delay between the retina and V4, i.e., approximately 100 ms for the average duration of the saccade plus the conduction time of approximately 65 ms, i.e., around 165 ms after the start of the saccade. It is only after this delay that V4 should produce positive gamma and present effective activity for the recognition of the content of the image present on the retina at the end of the saccade, i.e., at the beginning of fixation. It appears obvious that the start of the fixation at the end of the saccade is a priority period for information processing.

We have already pointed out that this period is preceded by pre-saccadic inhibition. The ‘fixation- -amplifier’ hypothesis (Lisberger and Nusbaum, 2000) has been demonstrated and occurs even in the dark (Rajkai et al., 2008), demonstrating that it is driven by a motor mechanism independent of vision.

### 5.4 Top-down processing and pre saccadic attention influence scanpath

We might think at first glance that when we open our eyes, the world appears to us as it is, and that each element of our visual environment acquires an equivalent representation. Confronted with an overwhelming quantity of visual information we have the impression of fluently understanding what we see. We are not conscious of the complex attentional selection that helps us to prioritize visual input (Hanning et al., 2023). This impression is purely subjective; our vision is highly selective, even in the absence of exploratory eye movement. We can indeed choose voluntarily to wait or not to wait for the appearance of an object which, respectively, increases or decreases the sensitivity to this object, making its appearance sharper or more blurred (Carrasco et al., 2004).

This attentional selection is realized by the scan path forming by a series of saccades performed toward relevant information of the visual environment. However, to achieve this real feat the brain must in a certain way know the target towards which the saccade must be directed, this is called pre-saccadic attention. It was demonstrated that pre-saccadic attention increased the maximal response, the visual sensitivity (Deubel and Schneider, 1996), the acuity (Kwak et al., 2023), and contrast sensitivity (Hanning et al., 2022) at the saccade target and reduced it at the non-target locations (Li et al., 2021).

The conscious experience of a scene depends both on the content of the retinal image and the state of attention of the brain. In this context, when several stimuli are present simultaneously in the visual field, they compete to achieve the most effective cortical representation (Desimone and Duncan, 1995).

This competition can be resolved by the voluntary deployment of selective attention which activates “top-down” signals which originate in the regions responsible for executive control and located at the level of the prefrontal cortex. The activation of neurons in the prefrontal cortex increases the firing frequency of neurons located at a lower hierarchical level and coding for the expected object.

However, not all processes managing attention are voluntary or under the control of a top-down process. For example, when you find yourself in a store waiting for someone to take care of you, each time the door opens entry, your attention will be drawn to it. This is another control process that can be described as “bottom-up.” In this case, attention is captured by particular stimuli that are intrinsically meaningful (salient) (Beck and Kastner, 2005).

The competition for a cortical representation is a consequence of the hierarchical architecture of the visual system: at the level of V1 (the primary area of the visual cortex), the receptive fields are very narrow (0.5 to 1.5 deg) and the behavior of the cells is simple, complex and hypercomplex boils down to local visual properties that are ultimately simple (compared to the complexity of the objects perceived) such as the recognition of contrasting bands and the orientation of the edges of objects (Hubel and Wiesel, 1998). Neurons in secondary and subsequent visual areas, such as V2, V4, and the inferotemporal cortex (IT), have larger receptive fields and more complex and highly controlled visual properties. Thus, V4 neurons have receptive fields whose diameter is approximately equal to the distance between the center of the retina and the fovea and respond to complex contours (Pasupathy and Connor, 2002). Neurons in area IT have large receptive fields that can span an entire visual hemifield and can respond to complex shapes and faces (Grill-Spector, 2003).

### 5.5 From eye movement recordings to brain generators identification

The advent of recent functional imaging using high-density EEG dynamics coupled with mobile eye-movement recordings may offer a synthesis of the different mental operations occurring since the onset of the pictural presentation, the dynamic of the eye scan paths exploration, the emotional reaction (detected by the pupil dilatation) (Waschke et al., 2019; Shourkeshti et al., 2023), and the final interpretation. Each of these functional operations can be detected and deeply explored using the event-related potentials (ERP) triggered by the onset of the picture presentation and for the different repetitive elements of the eye scan paths. Then for each ERP component, a frequency analysis of the EEG waves can be performed and quantified following the event-related spectral perturbation (ERSP) and the inter-trials coherence (ITC) (phase locking factor).

When these neuronal activities recorded on the scalp were defined, a deeper exploration can be made to explore the neural generators responsible for the genesis of the ERP and ERSP components. For this, independent component analysis (ICA) can be performed to select the ICA that originated in the brain from those coming from outside and not related to the studied processing. The neural generators of these ICA dipoles were localized in the brain and 3D inverse modeling methods (Makeig et al., 2002; Cebolla et al., 2011) were used to better define the localization of the generators corresponding to ERSP or ITC components occurring during the pictural exploration. Thanks to these operations a dynamic evolution of the different generators over time can be obtained and will help to better understand the electrical activities of the brain.

Finally, all of these different databases issued from high-density EEG dynamic can be introduced into different computational pipelines such Reimannian classifiers, convolutional neural networks, and deep learning (Simar et al., 2020, 2022). This methodology can be compared to the different models and computational neuroaesthetic approaches already proposed (Li and Zhang, 2020).

Human perceptual behavior can be initiated unconsciously or consciously, driven by curiosity, emotion and attention. The mental attitude of recognizing form through reduction, abstraction and elaboration is greatly based on learned automatisms and their appropriate inhibition, when necessary. Form and pattern recognition are based on criteria of global form, characteristics, but also on the architecture of their links.

## 6 Conclusion

In summary, the present proposal suggests the development of a new approach to dynamic scanpath EEG analysis aimed at enhancing expertise and connoisseurship in neuroaesthetics. This approach should be drawn from various perspectives established in psychology, neuroaesthetics and neuroscience, fostering a dialogue between psychometric and neurometric methodologies. This dialogue should be facilitated by novel experimental paradigms rooted in theoretical frameworks that consider the specific nature of neuroaesthetic inquiry about the relation between the viewer and the work of art.

The proposed method involves recording eye scanpaths and analyzing factors such as saccadic-blink suppression, fixational eye movements, sensory-motor mnemonic traces, saliency-meaning, and integrated priority maps. These analyses would be coupled with EEG recordings capturing different brain oscillations, which can aid in identifying distinct brain states and neural generators. These recordings would be conducted in carefully controlled artistic environments, allowing for a more precise definition of the neurophysiological signatures associated with the neuroaesthetic experience. Such an approach holds promise for the scientific understanding of the brain mechanisms involved in hedonic-aesthetic valence in cognitive perception, in the appreciation of quality, in expertise and in connoisseurship, especially in paintings.
